# MiFoDB, a workflow for microbial food metagenomic characterization, enables high-resolution analysis of fermented food microbial dynamics

**DOI:** 10.1128/msystems.00141-25

**Published:** 2025-08-19

**Authors:** Elisa B. Caffrey, Matthew R. Olm, Caroline I. Kothe, Hannah C. Wastyk, Joshua D. Evans, Justin L. Sonnenburg

**Affiliations:** 1 Department of Microbiology and Immunology, Stanford University School of Medicine 10624 , Stanford, California, USA; 2 Sustainable Food Innovation Group, The Novo Nordisk Foundation Center for Biosustainability, Technical University of Denmark 5205, Lyngby, Capital Region of Denmark, Denmark; 3 Chan Zuckerberg Biohub 578083, San Francisco, California, USA; 4 Center for Human Microbiome Studies, Stanford University School of Medicine 10624 , Stanford, California, USA; University of California Irvine, Irvine, California, USA

**Keywords:** nonhuman microbiome, fermentation, metagenomics, microbial communities

## Abstract

Fermented foods, which contain a diversity of microbes and microbial metabolites, have been used for millennia to increase food security, flavor, and nutritional content; more recently, they have been recognized as potential mediators of human health. Metagenomics is a powerful approach to characterize microbes in fermented foods, providing high taxonomic resolution and functional insights. Here, we introduce the Microbial Food DataBase, a metagenomics-based approach designed for the identification of fermentation-associated microbes. Using this primary database of metagenome-assembled genomes and relevant deposited genomes of prokaryotes, eukaryotes, and common food-relevant substrates, we investigated 89 fermented food samples. We present a streamlined high-confidence characterization of microbial diversity in fermented food, identifying previously undiscovered genomes and facilitating strain-level tracking across food environments. The easy and robust functionality of the workflow has significant implications for advancing food safety, promoting desired microbial communities, and increasing sustainability in food production.

**IMPORTANCE:**

Fermented foods have microbial communities that influence food safety, flavor, and human health. Microbial Food DataBase (MiFoDB), an alignment-based sequencing workflow and database, addresses the limitations of existing tools by enabling strain-level resolution, identifying novel genomes, and providing functional insights into microbial communities. Applying MiFoDB to fermented food samples, we demonstrate its ability to uncover novel species, track microbial strains across substrates, and integrate functional annotations. Additionally, the outlined workflow is highly customizable and can be used to generate alignment-based databases for other microbial ecosystems. This work highlights the importance of fermentation-specific workflows for studying microbial food ecosystems, advancing food safety, sustainability, and innovation in fermented food research.

## INTRODUCTION

Food fermentation has been used for thousands of years as a method of enhancing flavor, preserving, and promoting food safety, involving the microbial conversion of food components into desired food products (1). Microbes associated with food fermentation are of growing interest for their ability to convert waste streams into edible and healthy biomass toward more sustainable food systems (2). There is currently a rapid expansion of research into how microbial foods influence human immune and metabolic health (3–7), including global interest in combating malnutrition (8). This highlights the vast global diversity of fermented foods and underscores the increase in demand for the development of research tools tailored to fermented foods.

Sequencing technology has revolutionized our ability to identify microbes active in fermented foods, but many challenges remain. Marker gene-based methods (e.g., 16s rRNA and ITS sequencing) provide a census of microbes in food, but only at a coarse taxonomic resolution (approximately genus-level). Metagenomic sequencing has the ability to resolve species- and strain-level diversity of fermented foods, but methodological improvements are needed to fully leverage this technology. Most short-read taxonomic profilers today use clade-specific marker genes (e.g., MetaPhlAn4 [9]) or k-mer-based profiling (Kaiju [10], Kraken [11], and Clark [12]). These methods are computationally efficient, but the programs implementing these approaches generally have at least one of the following tradeoffs: (i) report percentage of mapped reads values scaled to 100%, preventing assessment of the amount of unclassified reads present in a sample; (ii) cannot report metrics like breadth of coverage to assess the confidence that a particular microbe is detected; (iii) do not provide assessment of microbial genes detected; and (iv) in practice are almost exclusively used with their provided reference databases, precluding detection of novel genomes and functional analysis. Alignment-based profiling methods, like inStrain (13), largely overcome these limitations, but are dependent on the use of a reference database and are computationally more intensive. While recent work has expanded MetaPhlan4’s database to include a large collection of fermented food metagenomes (14), a database for alignment-based sequencing is still lacking. The development of a metagenomic workflow for fermented foods with the ability to fully resolve species- and strain-level diversity would allow far more insight into the fermented food microbiota, something that would be especially informative for spontaneous ferments, which typically harbor extensive microbial diversity unknown to the fermenter and often unpredictable (15).

Here, we present Microbial Food DataBase (MiFoDB), a workflow for the identification of prokaryotic and eukaryotic genomes in fermented foods, with an accompanying customizable database. This workflow overcomes all the above-listed shortcomings by leveraging full genome characterization, providing high accuracy in genome detection, ability to detect novel genomes, strain-level resolution and tracking, and functional insight into microbial communities. Application of the full MiFoDB-based metagenomic workflow easily allows other users to conduct strain-resolved, functional analysis of their fermented food metagenomes. As interest in food fermentation grows, gaining a high-resolution and comprehensive view of microbes involved in distinct fermentation processes will help improve food safety, quality, and nutritive value (1, 16). This MiFoDB-based metagenomic workflow easily allows other users to conduct strain-resolved, functional analysis of their fermented food metagenomes, facilitating novel microbe discovery and mechanistic studies in the growing fermented food research landscape. A full description of the MiFoDB workflow and accompanying open-source database is available in GitHub (https://github.com/elisacaffrey/MiFoDB), and documentation is available online at https://mifodb.readthedocs.io/en/latest/.

## RESULTS

### Metagenomic sequencing of a diverse collection of fermented food samples

**Fig 1:**
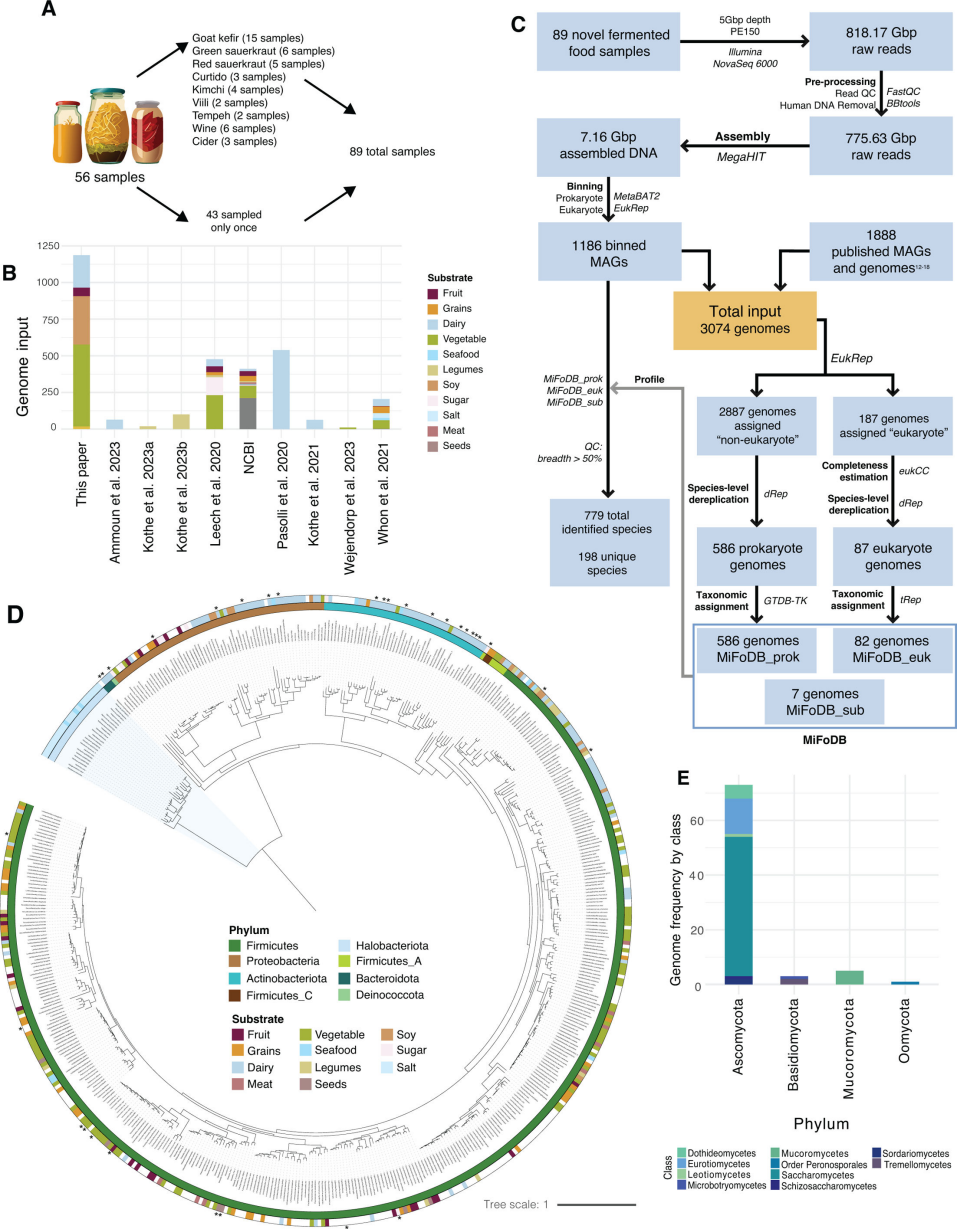
MiFoDB workflow and genome database generation. (A) Overview of sample collection for shotgun metagenomic sequencing of fermented food samples. (B) Summary of all input genomes by each genome substrate origin, including MAGs assembled in this paper, previously published MAGs, and reference genome. (C) Workflow for metagenome recovery and MiFoDB development. A total of 775.63 Gbp of data were generated from all 89 samples (range = 0.12–26.14 Gbp; avg = 8.7149 ± .63 Gbp SEM). (D) A phylogenetic tree of prokaryotic genomes is included in the final MiFoDB_prok reference. The tree is decorated by phylum (inner ring) and substrate of origin for each reference genome (outer ring). Archaeal genomes are highlighted with the blue background. Novel genomes are denoted with an asterisk. (E) Frequency of the eukaryotic genomes is included in the final MiFoDB_euk reference by phylum; shade indicates class. A member of order Peronosporales does not belong to an assigned class and so is listed by order.

Characterizing the microbial landscape of foods remains an important challenge to understand which microbes play a role in fermentation and how they interact within their community to influence food safety, flavor, and human health. Here, we performed metagenomic sequencing on 56 fermented foods, collected between January 2021 and March 2023 (Table S1). From these 56 ferments, the majority of which were vegetable ferments, we sequenced 89 samples in total, including various time points and from different parts of the food (Fig. 1A). Across all samples, we recovered a total of 1,186 microbial genomes (see Materials and Methods).

### Generation of the MiFoDB genome database

Recognizing how commonly used databases, not tailored to fermented food environments, might miss food fermentation-specific genomes, we leveraged the above-described sequencing to create a novel, custom fermented-food-focused metagenomic alignment-based database. Alignment-based profiling, which quantifies the breadth of genome coverage, allows for high-confidence detection of low-abundance microbes. The inputs to this database included the 1,186 microbial genomes recovered in this study and 1,888 previously recovered microbial genomes from fermented foods (Fig. 1C; Table S2) (see Materials and Methods). This genome set represents an up-to-date sampling of fermented food microbes, with a wide global distribution of sample origin from which genomes were derived (Fig. S1A) and genomes originating from dairy and vegetable ferments (Fig. 1B) especially prevalent in the total input set of 3,074 genomes (Fig. 1C, orange box).

We next selected representative genomes for each species-level group present in our database to overcome issues associated with mapping to multiple closely related genomes simultaneously (13). We opted for a tripartite database strategy, featuring MiFoDB_prok for prokaryote identification, MiFoDB_euk for eukaryote identification, and MiFoDB_sub for food substrate identification (Fig. 1C). This modular approach allows for the use of domain-specific thresholds, higher accuracy in gene calling, and targeted downstream analyses. By implementing a dereplication pipeline (see Materials and Methods), we ultimately identified 586, 82, and 7 species-level representative genomes in the prokaryotic, eukaryotic, and substrate databases, respectively (Table S3).

The final MiFoDB_prok database contains 586 genomes, representing 558 bacterial and 28 archaeal genomes (Fig. 1D; Fig. S1B). Bacterial genomes within MiFoDB are dominated by Firmicutes, primarily of the family *Lactobacillaceae*, reflecting the dominance of the lactic acid microbe reference genomes. While archaeal genomes were identified in sequenced salt used in kimchi making or seafood ferments, bacteria reflected a wider distribution of substrates (Fig. 1D). Of note, no taxonomic identification was assigned at a species level to 49 bacterial genomes (Table S4; Fig. S2A). These novel MAGs originated from eight different countries (Fig. S2B through D). Expansion of this database is expected with future sequencing of novel samples from diverse geographic regions. In addition, eight genomes representing unique species were noted as likely misannotated in NCBI, seven of which are putative novel species and have been included in the list of novel genomes (Table S4).

The final MiFoDB_euk database contains 82 representative eukaryotic genomes. Following taxonomic identification of these genomes, five represented substrate genomes (Table S5) rather than microbial eukaryotes involved in fermentation and were subsequently removed from the MiFoDB_euk database, resulting in the 82 eukaryote members in the current MiFoDB_euk (Fig. 1E; Table S1). Four eukaryotic genomes were annotated as *Kazachstania saulgeensis* but with >95% ANI, indicating these are likely to represent three or more novel close relatives of *K. saulgeensis*.

Finally, for the identification of substrate genomes in the metagenome sample, MiFoDB_sub was developed. The database consists of seven high-quality RefSeq genomes from *Brassica oleracea *var*. oleracea* (wild cabbage plants)*, Bos taurus* (cow), *Capra hircus* (goat), *Vitis vinifera* (wine grapes), *Glycine max* (soybean), *Oryza sativa* (rice), and *Triticum aestivum* (common wheat) (Table S3).

### MiFoDB allows for detailed insight into the fermented food microbiota

**Fig 2:**
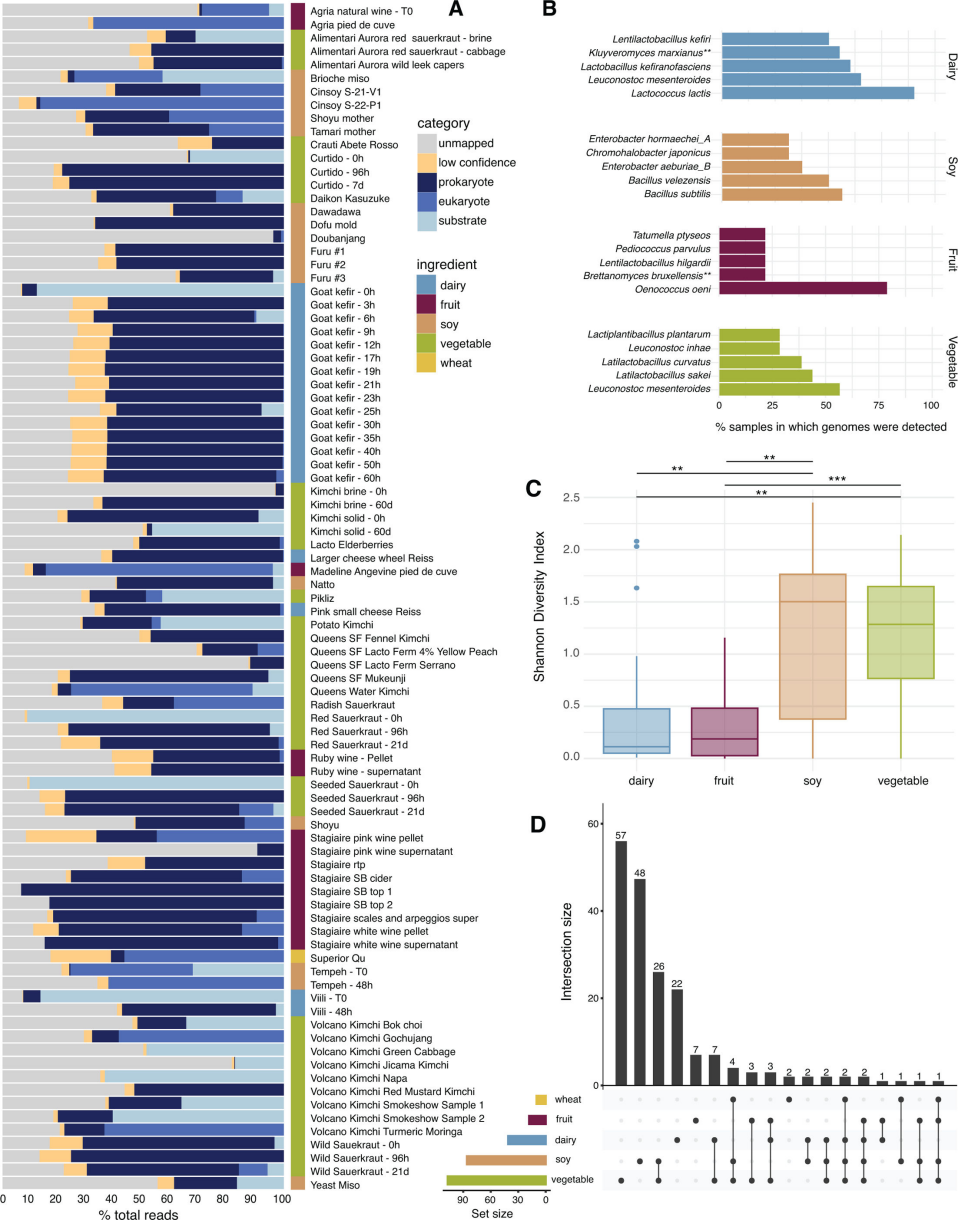
Mapping of fermented food samples using MiFoDB. (**A**) Profiling results of fermented food samples using MiFoDB, mapped to prokaryotic, eukaryotic, or substrate (mean reads mapped = 62.62% ± 2.08% SEM of reads). Low-confidence profiling results (breadth < 0.5) and unmapped reads are also noted. (**B**) Bar plot of the five most frequently identified microbes by substrate category. Eukaryotes are differentiated with (**). Wheat samples are not included, as only one wheat-based ferment was sequenced. (**C**) Shannon diversity index of microbes (eukaryotic and prokaryotic) profiled by ingredient category. *P* values from Tukey’s test; *P* < 0.05 (**), *P* < 0.001 (***). Wheat samples are not included, as only one wheat-based ferment was sequenced. (**D**) UpSet plot of intersecting species across ingredient types. The bar plot above represents the number of total species within each set. The rows below each represent an ingredient, with the edges connecting nodes that are included in the set. The bar plot on the left shows the total number of species for each substrate. No species was identified in all four ingredient categories.

We next applied our tripartite MiFoDB database (prokaryotic, eukaryotic, and substrate) to profile all ferment metagenomes sequenced at the start of this study (see Materials and Methods). The MiFoDB database recruited the majority of reads in most samples (mean reads mapped = 62.62% ± 2.06% SEM) (Fig. 2A). These values are consistent with mapping being influenced by community diversity observed for other environments such as soil microbiomes (low percent of reads mapping) (17–19), and infant gut microbiome (high percent of reads mappings) (20, 21). In a few samples, the substrate genome mapped to up to 91% of the total sequenced metagenome. Inclusion of substrate reference genomes allows for a deeper understanding of the fermented food profile. Common bioinformatics methods normalize the total of all microbial mapped reads to 100%, disregarding the portion of unmapped reads, which in some cases represent the majority of sequencing data. This normalization method provides a clear picture of the relative abundance of different classes of reads to total mapped reads, but skews our understanding of microbial diversity and novelty in the sample (Fig. S3A). Ignoring the portion of reads that remain unmapped limits insight into uncharacterized microbial (or substrate) content. To address unmapped reads, we profiled all our samples against GTDB (22) using sylph (23) (Note S1).

MiFoDB reflects the expected microbial content of fermented foods for the different substrates (Fig. 2B; Table S6a) (24–30). In the dairy samples, *Lactococcus lactis* was present in 90% of dairy-based samples, along with *Leuconostoc mesenteroides*, *Lactobacillus kefiranofaciens*, *Lactobacillus kefiri*, and the yeast *Kluyveromyces marxianus,* reflecting the high kefir sample representation in our data. Soy and vegetable substrate ferments had higher diversity (Fig. 2C), with *Bacillus subtilis* and *L. mesenteroides* present in just over 50% of samples, respectively. *Oenococcus oeni*, involved in malolactic fermentation, was observed in >78% of fruit (wine) samples, highlighting the dominance of the microbe at the final stages of wine production. Comparing the presence of microbes across substrates, vegetable ferments had the greatest number of unique genomes (Fig. 2D).

### MiFoDB enables exploration of understudied fermented foods

To evaluate MiFoDB’s ability to characterize understudied fermented foods, we took a closer look at superior qu, a poorly characterized wheat-based ferment. Used as a starter in a variety of fermented foods, including alcoholic beverages and jiangs, and produced by spontaneous fermentation of wheat berries, understanding the microbial complexity of this ferment gives insight into the flexibility of its use. To highlight the importance of a microbial food-specific database, profiling with inStrain was performed using either UHGG (31), the Unified Human Gastrointestinal Genome, or MiFoDB. For additional comparisons, Kaiju (10) and Kraken2 (11) were used as k-mer based profiling methods, while MetaPhlAn4 (9) was used as a clade-specific marker gene based profiling method (Fig. S4 and S5).

**Fig 3:**
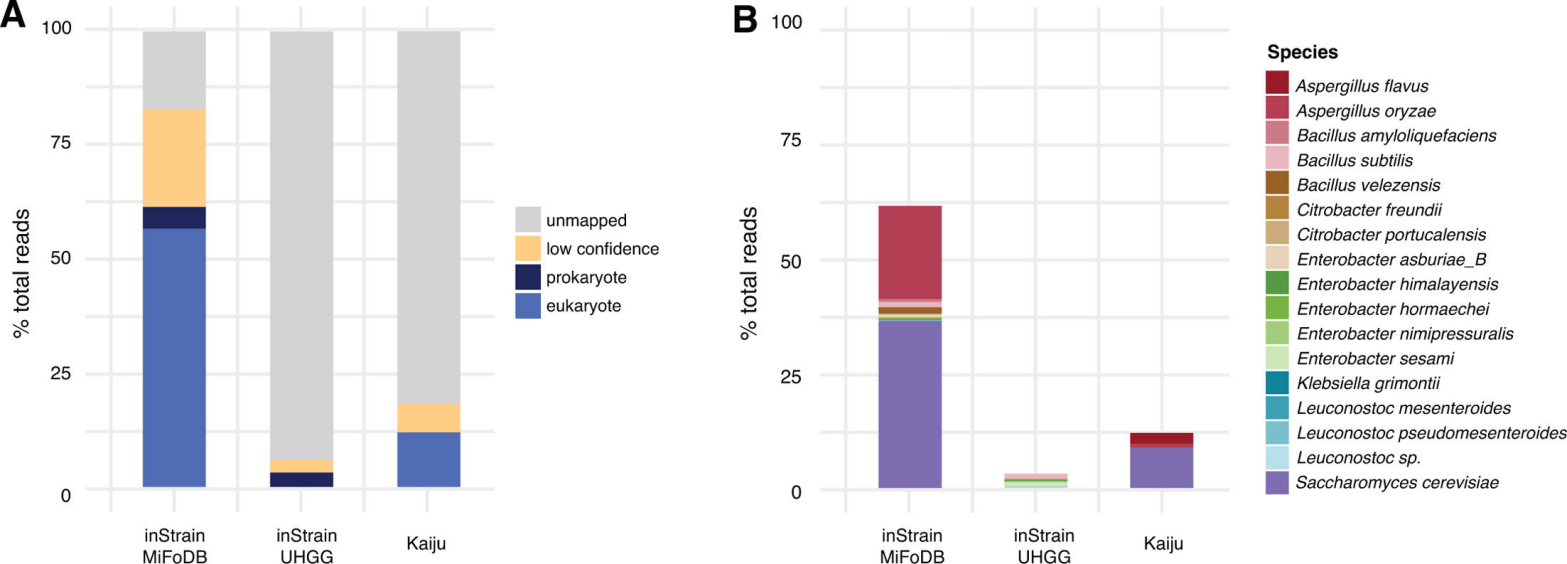
Comparison of alignment-based metagenomic profiling databases. (**A**) Comparison of the percentage of total reads using MiFoDB, UHGG, and Kaiju for superior qu. Mapped reads include identified prokaryotes and eukaryotes, as well as low-confidence reads mapped (breadth < 0.5) and unmapped reads. In all, 62.62% of reads mapped with high confidence to MiFoDB versus 3.12% for UHGG. Kaiju reported 12.0% high-confidence read mapping and only reported the presence of three eukaryotes in the sample. (**B**) Percent of high-confidence mapped reads (breadth > 0.5 for inStrain and abundance > 1 for Kaiju) by species designations using MiFoDB, UHGG, and Kaiju. Using a fermented food-focused database increases read mapping by 49–58%, with a variety of food-relevant eukaryotes and prokaryotes.

Using inStrain, we first compared the mapping performance of MiFoDB versus UHGG; since Kaiju, like inStrain, reports low confidence and unmapped reads but uses a separate database, we included it as a reference. Profiling results for the superior qu revealed the better performance of MiFoDB over both UHGG and Kaiju (62.6% high-confidence mapped reads vs 3.12% and 12.0% respectively; Fig. 3A). Comparing species identified using these three methods (Fig. 3B) revealed that UHGG lacked two of the most abundant eukaryotes identified using MiFoDB, *Aspergillus oryzae* and *Saccharomyces cerevisiae*. Kaiju only reported high confidence in the identification of three eukaryotes: *S. cerevisiae*, *A. oryzae,* and *Aspergillus flavus*, a pathogenic fungus known for aflatoxin production (32).

Differentiating between *A. oryzae* and its aflatoxin-producing relatives is of clear importance for food safety (Note S2; Table S7). MiFoDB reported high-confidence identification of both *S. cerevisiae* and *A. oryzae*, a yeast and a filamentous fungus, along with a number of bacteria previously associated with fermentation, supporting the wide substrate applicability of this starter. The larger microbial profile identified using MiFoDB compared to UHGG highlights the need for a fermented food-focused metagenome reference database, while compared to other profiling methods (Fig. S4 and S5) showcase the value of alignment-based profiling in high-confidence genome profiling, particularly in understudied fermented foods.

### Diverse fermented foods share identical starting strains

**Fig 4:**
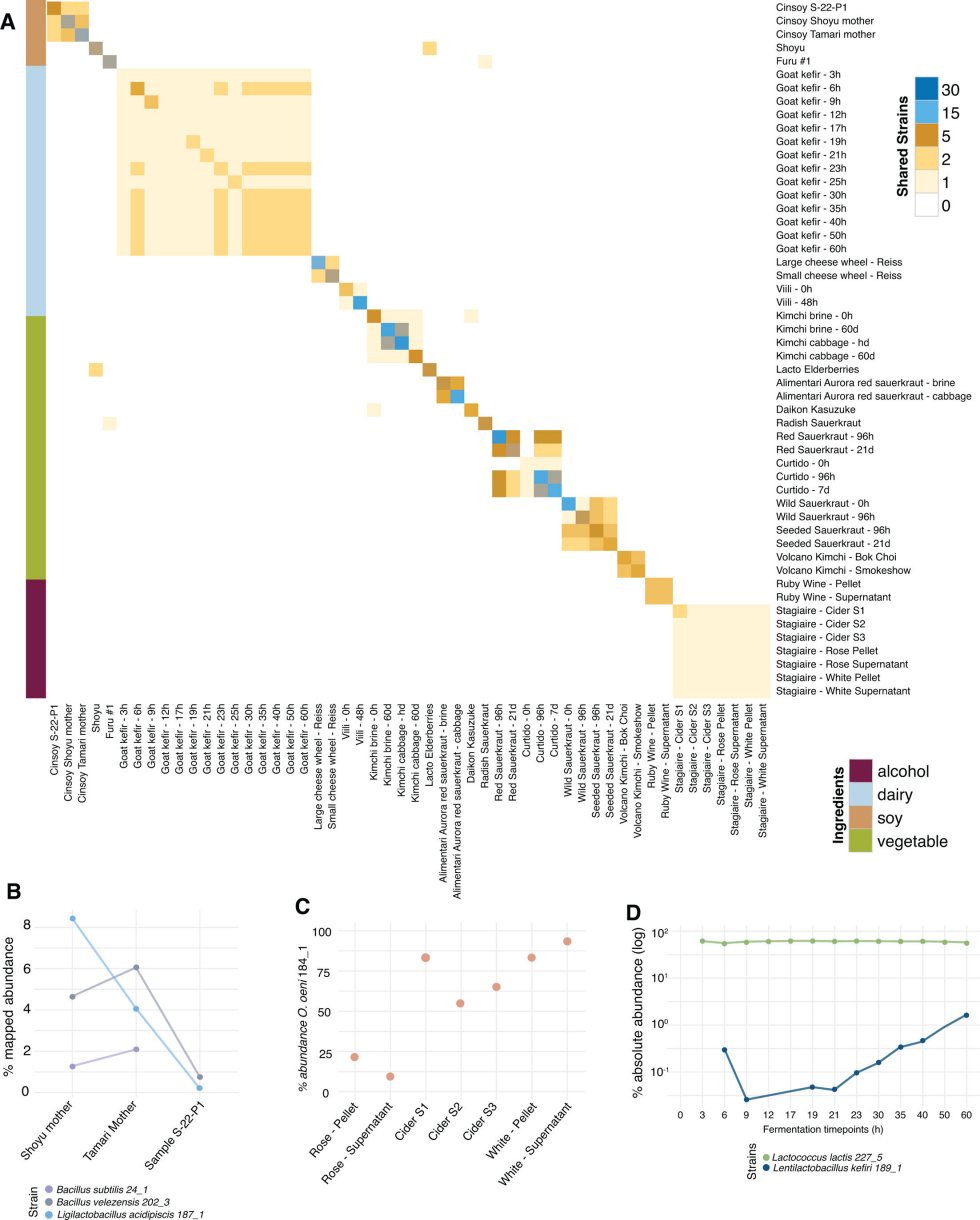
Strain sharing across fermented food samples. (A) inStrain-determined strain sharing between samples. Strains are primarily shared between related samples (same samples across different time points, samples made by the same producer, and samples used as a starter for a second sample). (B) Strains tracked between starters (Shoyu mother and Tamari mother) and packaged product (sample S-22-P1). (C) One strain of *Oenococcus oeni* tracked across samples of cider and wine made without added starters by the same producer at different time points. Three samples of the same cider, a pinot noir rosé, and a sauvignon blanc and chardonnay blend, with fruit deriving from different geographic areas and produced across the span of a few months shared the same strain of *O. oeni* 184_1, likely derived from the shared fermentation environment. (D) Two strains tracked in goat kefir across 14 time points

Some fermented foods, like sourdough starters, are thought to have long-term colonization of particular flavor-imparting microbial strains (33). However, most culture-based or marker gene-based methods cannot differentiate between closely related strains, and thus cannot measure long-term persistence of microbial strains compared to continual extinction and rise of new strains within the starter. An important advantage of the MiFoDB-based workflow is the ability to perform unbiased and accurate identification of strains shared across samples and over time (Fig. 4A; Table S8). This includes strains shared through passaging, in ferments made by the same producer, and across time points.

By applying this method, strains of *Bacillus velezensis* 202_3 and *Ligilactobacillus acidipiscis* 187_1 were identified in both the shoyu and tamari starters (termed “mothers”), as well as the ferment produced after adding the starter (Fig. 4B). Strains can also be tracked across ferments made at different time points by the same producer (Fig. 4C). During milk kefir fermentation, *L. lactis* 227_5, which was likely present but below sequencing detection limit at the initial time point, reached close to 50% of total reads in the sample and was easily identified at all following time points, remaining at a similar abundance throughout the fermentation. *L. kefiri* 189_1 was not identified before 6 h, but increased steadily in abundance from 21 h to the final fermentation time point (Fig. 4D).

Multi-omics application of MiFoDB to strain-level metabolite profiles allows for a deeper interrogation of strain-specific microbe–substrate interactions and has important implications for food safety, taste profiling, and customization of fermented products. Characterizing the metabolite profile at a strain level has the potential to aid in the selection of specific strains in food production. Coupling semi-targeted metabolomics (Table S9) of the same milk kefir time-course series sequenced previously (Table S1) to the metagenomics strain results, we observe strain-specific metabolite signatures (Fig. S8). For example, the positive correlation between *L. kefiri* 189_1 and histamine abundance highlights the need to identify potential regulatory mechanisms of histamine production at a strain, species, or community level, mitigating histamine production and creating a kefir that is appropriate for those with bioamine sensitivities.

### MiFoDB reveals functional differences in the microbiota between fermented foods

**Fig 5:**
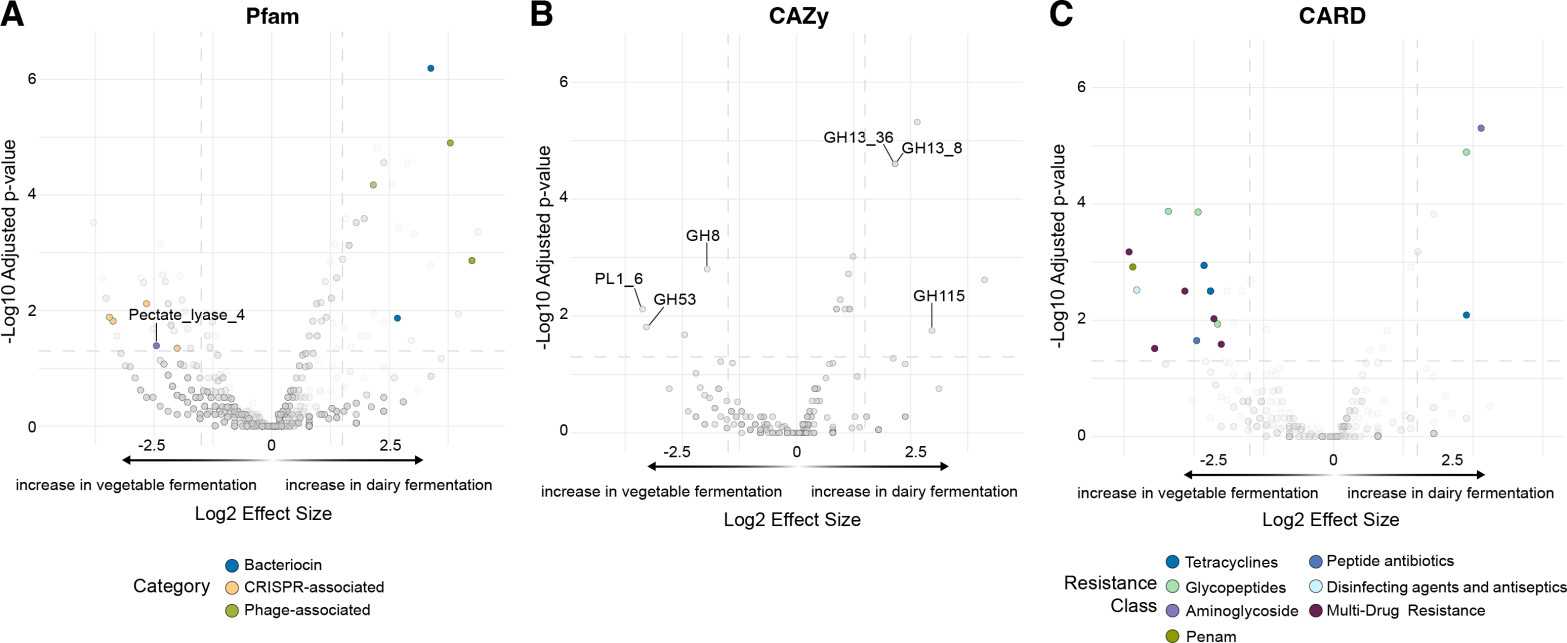
Increased representation of gene families in either vegetable or dairy ferments based on Pfam domains (**A**), CAZymes (**B**), or antibiotic resistance using the Comprehensive Antibiotic Resistance Database (CARD) (C). Genes with adjusted *P* values < 0.05 and log2 effect size of either 2 (A, C) or 1.5 (B) are colored with functional categories noted below each plot.

A key advantage of our MiFoDB workflow is its ability to directly profile microbial functional potential. Genes identified in our samples were annotated against Pfam (34) (protein family), CAZy (35) (carbohydrate-active enzymes [CAZymes]), and CARD (36) (antibiotic resistance genes) databases (Table S10a through c). Using fermented dairy (dominated by kefir samples) and vegetable ferments in our sample to illustrate this application, we found that CRISPR-associated domains (Cas1, Csn2, Cas9 PI, Cas2, and HNH4) were enriched in vegetable ferments, while dairy ferments were significantly associated with phage-related genes (L_lac_phage_MSP, Lac_bphage_repr, Phage_Treg, TerB-C, TerB-N, CW_7) and bacteriocin expression (Lactococcin and Helveticin-J). Pectate lyase 4, an enzyme involved in maceration and rotting of plant tissue, was significantly associated with microbial vegetable fermentation, but was depleted in dairy fermentation (Fig. 5A). The CAZy database identified glycoside hydrolase family 8 (GH8), formerly known as a member of the cellulase family, as enriched in vegetable ferments. Vegetable ferments show enrichment in pectate lyase (PL1_6) and GH53, an enzyme with specific activity on type I arabinogalactans, carbohydrate components of the primary cell walls of dicots (37). This finding, along with similar results from Pfam, highlights the importance of microbial pectin degradation in facilitating the fermentation of fermented foods, which is consequential for vegetable fermentation quality and product desirability.

Dairy ferments were enriched for amylases (GH13_8 and GH13_36) and Glycoside Hydrolase Family 115 (GH115), which includes α-glucuronidase activity (Fig. 5B), when compared to vegetable ferments. Profiling against the CARD database (Fig. 5C) identified a number of antibiotic-resistant annotations, with vegetable ferments enriched for Multi-Drug Resistance (MDR) genes when compared to dairy. The presence of antibiotic resistance, a naturally occurring defense mechanism in competitive microbial communities, has been previously discussed in fermented foods, particularly in lactic acid bacteria (28, 38–40). However, drug-resistance acquisition and its impact on health from fermented food-associated microbes require further study and contextualization with other microbial communities such as those from the human gut microbiome.

## DISCUSSION

As scientific inquiry of fermented food continues to grow, appropriate tools are needed to characterize complex interactions of their microbial communities. This study offers an assembly-based workflow and database to profile the fermented food metagenome. With over 600 medium- to high-quality genomes of prokaryotes, eukaryotes, and common fermented food substrates, our approach allows for high-confidence identification of microbes underrepresented in other databases. While the database size of identified MAGs remains a limitation, the customizability encourages the incorporation of novel and higher-quality food-associated MAGs and substrate genomes, facilitating the investigation into microbial diversity, strain-tracking, and functional analysis for any food of interest, especially understudied fermented foods. One of the disadvantages of alignment-based profiling remains the increase in runtime compared to other profiling methods (13, 41). However, the advantages of this profiling method include the ability to assess the unclassified reads and microbial genes, advantages that in many cases will outweigh the runtime limitation, as discussed in the introduction. Guidance on run time considerations and parameter optimization is available on MiFoDB.readthedocs.io.

Our analysis of 56 fermented foods supports previous work on the ferment metagenomic landscape (14, 28, 42), including recent work by Carlino et al. 14. Of note, soy and vegetable fermented foods shared 26 unique species, and compared to other fermented food substrates, showed higher species- and strain-level resolution, breadth as a coverage metric, and diversity (Fig. 2D). These shared species include lactic acid producers commonly observed in vegetable-based fermented foods like *Lactiplantibacillus plantarum*, *L. mesenteroides*, and *Weissella soli* (43–46), as well as several *Klebsiella*, *Enterobacter*, and *Staphylococcus* species, each identified mostly at <5% of total reads (Table S6a) likely due to carry over from the soil and production environment (44) (including the producer) prior to preparation for fermentation. Their continued presence in fermented food might be due to extrinsic factors affecting community assembly based on substrate-specific ferment preparation. This higher diversity highlights the potential importance of low-abundance microbes in the microbial fermentation community. Characterizing lower-abundance microbes can be an important tool in modeling community assembly (47, 48), understanding their role in food safety and flavor development (15, 44, 49, 50), and exploring the role of fermented foods in health (3, 6, 51), facilitated by our database and workflow.

It is estimated that there are hundreds of fermented foods around the globe (52, 53), and more microbial foods will be created through sustainability projects (2, 44, 54). With the increasing availability of low-cost sequencing, metagenomics is already becoming the standard method of characterization of these foods, and MiFoDB can support the identification and incorporation of novel microbes into the database. Consistent with Carlino et al., our analysis revealed 49 novel prokaryotic species, of which 25 species were identified from dairy samples, primarily cheeses, underscoring the diversity in well-studied categories of fermented foods. Using GTDB to identify this novelty emphasizes how novel genomes reported in this study are not just unique to fermented foods but are novel across all available deposited prokaryotic genomes. While these microbes remain to be isolated and characterized, inclusion in MiFoDB facilitates cutting-edge investigations.

While efforts are ongoing to support research in food fermentation outside of the United States and Europe (8, 14, 28, 55, 56), many of the fermentation-associated microbes and genomes have been identified primarily in ferments from industrialized countries. Expanding research support for scientists outside the United States and Western Europe, as our database and workflow aim to do, will hopefully lead to a more complete understanding of the global fermented food microbial landscape.

To streamline functional and strain investigation of fermented food-associated microbes, integration of MiFoDB into the alignment-based taxonomic profiling program inStrain offers additional advantages. First, performing functional analyses with tools like CAZyme and CARD informs our understanding of attributes like metabolic activity and drug-resistance profiles. Second, microbial strains that represent less than 10 years of evolutionary distance between two members of the same species (13) can be tracked across samples in an unbiased manner. While we show a number of examples of strain tracking, MiFoDB can be used to track microbial strains from their environmental source to the fermented food and across time points, addressing questions of strain origin, evolution, and microbe–host relationships. Correlating metabolite production with strain abundance supports the investigation of strain-specific phenotypes, which will enable ferment customization based on desired metabolite properties. For example, we observed *L. kefiri* 189_1 was positively correlated with histamine production, while *L. lactis* 227_5 was positively correlated with ethyl maltol production, a compound with a caramel flavor (57). Using MiFoDB to differentiate strain-specific phenotypes facilitates strain selection or community customization in a fermented food to decrease the production of undesired metabolites like histamine or increase the production of molecules beneficial for flavor or health. This would allow for the personalization and contextualized innovation of fermented foods, such as optimizing fermentations on new food substrates for sustainability. In addition, functional characterization of novel food-associated genomes supports innovation in culinary, food safety, biomedical, and sustainability efforts.

Application of MiFoDB can address a number of questions. For example, MiFoDB can be used to identify species that are typically safe, but recognized as having opportunistic pathogen potential, such as *Enterococcus faecalis* (58). Monitoring the presence and load of such microbes will add to our understanding of whether such microbes pose any health concerns for specific populations (e.g., immunocompromised individuals, the elderly). With the growing interest in developing bespoke microbial communities for food fermentation (59), MiFoDB supports the characterization of a community at a strain level. Coupled with functional analysis and metabolomics, proteomic, or other phenotype metrics allows researchers to understand how strain-specific presence, absence, or abundance impacts a measurable outcome. This has broad application not only for microbial communities as model systems, but also for culinary (e.g., production of a specific flavor), and health (ensuring presence or absence of a specific metabolite of interest) applications. In addition, the application of MiFoDB to characterize fermentation of novel substrates, like waste products from food production, will enable a reliable and rapid understanding of the communities that can be cultivated using underutilized substrates for sustainability applications (2).

MiFoDB presents a streamlined workflow for the high-confidence identification and profiling of microbes involved in food fermentation, coupled with tools for functional analysis and strain tracking. Its customizable database can be expanded to include any food-relevant prokaryotes, eukaryotes, and substrate genomes. In fact, the latest updated version of the database, already publicly available, incorporates novel MAGs (14) published during the revision of this paper. By reporting both the mapped and unmapped reads in a sample, it presents high-confidence results and highlights microbial novelty. Integration of functional analyses and strain tracking offers powerful tools for researchers investigating food fermentation from microbiology to culinary work. Application of MiFoDB to both novel and previously well-characterized fermented foods will help illustrate how substrate and preparation techniques influence microbial communities. Detailed microbial and molecular characterization of fermented foods will help us better understand how fermentation practices, both modern and traditional, influence safety, health, and flavor.

## MATERIALS AND METHODS

### Fermented sample preparation

Time course samples of kefir, curtido, kimchi, viili, and sauerkraut were prepared between 2021 and 2022. Milk kefir was fermented as previously described (29). Briefly, 1 L of goat milk was inoculated with 60 g of kefir grains and fermented for 60 h at room temperature. Curtido was prepared with 25% carrots, 15% onions, 4% green onions, 4% jalapenos, and 52% green cabbage by weight, all sliced with 2% salt added. After sitting for 30 min, curtido was jarred and fermented for 7 days before moving to the fridge for longer-term storage. Both red and green sauerkraut were prepared by thinly slicing red or green cabbage and adding 2% salt by weight. The initial time point sample was collected before letting the salted cabbage sit for 1 hour prior to jarring. For the preparation of the “seeded sauerkraut,” green cabbage was prepared as described. However, just prior to jarring, 5% of cabbage by weight was removed and replaced with equal weight of a commercial green cabbage sauerkraut prepared with only cabbage and salt. Sauerkrauts were fermented for 21 days at room temperature in a jar with an airlock, prior to refrigeration. Kimchi was prepared using the standardized method obtained from the World Institute of Kimchi (60) of 5% Korean red pepper flakes, 3% garlic, 2% salt, 35% water, 1% sugar, 4% fish sauce, and 50% napa cabbage by weight. Napa cabbage was washed and salted with 4% salt by weight prior to rinsing and mixing with paste and jarring. Kimchi was fermented in a self-venting crock for 14 days at room temperature before being moved to 4°C for long-term fermentation up to 60 days. Viili was prepared by mixing 500 mL of cow milk with the viili starter and incubated at room temperature for 48 h. All samples were collected pre-inoculation and at set time points before being immediately frozen on dry ice and stored at −80°C prior to analysis. Superior qu was prepared by a fermenter in Boulder, CO, USA (Table S1), and sampled in Liberty, TN, USA, based on a recipe from Qinmin Yaoshu dating to 540 CE. For the sample preparation, equal parts of wheat berries were steamed, roasted, or ground raw and mixed with water to form a stiff paste, shaped into cakes, and left to dry on a mat. Every 7 days, cakes were flipped; on day 7 cakes were stacked and placed in a jar with a lid for 7 days, and finally hung until completely dry.

### Sample collection

Fermented food samples were collected between 2021 and 2023, and sample source was reported (Table S1). Fermented food included in this study was collected based on our own or collaborator access to the samples. To fill a gap in data and knowledge about fermentation microbiomes, we focus our sequencing efforts on spontaneous ferments (those that arise *de novo* and those that are propagated via backslopping). All samples were either collected directly into Zymo Research DNA/RNA Shield Lysis Tubes, or frozen on dry ice immediately upon collection and stored in −80°C prior to DNA extraction.

### Library preparation and sequencing

Shotgun metagenome sequencing was performed on DNA extracted using ZymoBIOTICS DNA Miniprep Kit. For samples that do not specify a sampling area (brine or solid), a composite was taken. All samples were then bead beaten following ZymoBIOTICS DNA Miniprep instructions, and extraction was performed as recommended. For samples EBC_096 through EBC_114 (Table S1), libraries were prepared and sequenced as previously described on a NovaSeq 6000 using S4 flow cells at Chan Zuckerberg Biohub (San Francisco, CA, USA) (21). All other sample libraries were prepared using Roche KAPA HyperPlus and 96-UDI plates following the manufacturer’s instructions. Pair-end sequencing (2 × 150 bp) was performed using Illumina shotgun sequencing at the UC San Diego IGM Genomics Center utilizing an Illumina NovaSeq 6000 that was purchased with funding from a National Institutes of Health SIG grant (#S10 OD026929). In total, 818.17 Gbp of raw sequencing were generated.

### Metagenome quality control and assembly

Raw sequencing was demultiplexed and processed using BBtools (BBMap; B. Bushnell, sourceforge.net/projects/bbmap/), including marking of exact duplicate reads (clumpify), trimming of adapters and low-quality bases (bbduk; trimq = 16, minlen = 55), duplicate reads were removed, and finally mapped against the human genome (hg19) to remove any human reads while maintaining broad eukaryote regions. FastQC (https://www.bioinformatics.babraham.ac.uk/projects/fastqc/) was used for read quality. Metagenome assembly was performed using MegaHIT v1.2.9 (61) and binned using MetaBAT2 v2.15 (62). In total, 1,186 novel bins assembled from the 89 samples (Fig. 1C) were included in the input data set, described below, generating 7.21 Gbp of read pairs.

### Prokaryote database development

Eukaryotic assemblies were eliminated as a first step in developing the database consisting of bacterial and archaeal genomes. EukRep-0.6.7 (63) was used across all assemblies to identify potential eukaryotes. Genomes with >50% eukaryote assignment and a total eukaryotic genome length of >6 Mbp were assigned as “eukaryotic.” dRep v3.4.5 (64) was employed on all assemblies that did not meet the criteria, which were assumed prokaryotic to determine species-level groups. Species groupings were based on ≥95% average nucleotide identity (ANI). Using the dRep dependency checkM v1.2.2 (65), completeness and contamination scores for all genomes were calculated. A quality threshold was set at >50% completeness and <10% contamination for the identification of “medium” quality genomes (66). dRep clustering identified 586 unique representative genomes, with 505 genomes (>86%) at >90% completeness and <5% contamination (Fig. S1C). All genomes were concatenated into a .fasta file, and a scaffold was made using parse_stb.py (from dRep v3.4.5 [64]) for the final MiFoDB_beta_v1_prok database. Taxonomic classification was assigned using gtdbtkv2.3.0 (67). Any genomes added from NCBI maintained their assigned taxonomy. Metagenomes with no assigned NCBI taxonomy and no species-level taxonomic classification using gtdbtkv2.3.0 were defined as “novel.” The majority of genomes serving as representatives for each species came from NCBI (318/586 genomes), reflecting the higher genome quality of deposited isolated reference genomes compared to *de novo* assembled MAGs (Fig. S1D). Identification of protein-coding genes was performed using Prodigal 2.6.3 (68). GtoTree (v1.7.00) (69) was used to make a phylogenetic tree with bacterial and archaeal gene sets and visualized using iTol (70) with taxonomy provided by gtdbtk, using all default settings.

### Eukaryote database development

Assemblies that met criteria for “eukaryote” assignment (>50% eukaryote assignment and >6 Mbp genome length) using EukRep-0.6.7 (63) were then scored for completeness and contamination using eukCC ver_1.1 (71). Representative genomes were identified using dRep v3.4.5, with a completeness threshold >50% (-comp 50). As the contamination score of a number of NCBI reference eukaryotic genomes was >10% (despite being reference-quality isolate genomes), the contamination weight was set to 0 (--contamination_weight 0). dRep clustering identified 87 unique representative genomes. Protein alignment using DIAMOND (72) was performed against eukCC results and compared against UniRef100 (73) with a max *e* value of 0.0001. Taxonomic identification of genomes without assigned taxonomy from ODFM or NCBI was performed using tRep (https://github.com/MrOlm/tRep/tree/master/bin). tRep results annotated five assembled genomes as likely substrate genomes and unrelated to microbial fermentation (Table S5). These genomes were filtered out, and the final 82 genomes were used in the development of the final MiFoDB_beta_v1_euk database scaffold and fasta file.

### Substrate database development

For MiFoDB_sub, reference genomes from common fermented food substrates, including *B. oleracea* var. *oleracea* (wild cabbage plants), *B. taurus* (cow), *C. hircus* (goat), *V. vinifera* (wine grapes), *G. max* (soybean), *O. sativa* (rice), and *T. aestivum* (common wheat) were downloaded directly from NCBI. Genomes were concatenated into one .fasta file and scaffold file (parse_stb.py) to make MiFoDB_beta_v1_sub.

A concatenated fasta and scaffold-to-bin file was generated for all species-representative prokaryote genomes, eukaryotic microbe genomes, and substrate genomes. Files are available on Zenodo (https://zenodo.org/records/10870254).

### Metagenome mapping and diversity characterization

Reads from all trimmed fastq metagenomes were profiled using inStrain profile (inStrain v1.8.0 (13). Percent of total reads (% total reads) refers to the number of reads mapped to a specific species compared to the total number of sequenced reads post-adapter trimming and QC (described above in Metagenome quality control and assembly). Percent of mapped reads (% mapped reads) refers to the number of reads mapped to a specific species out of all the total reads mapped to any species. Percent of mapped reads was calculated based on # reads mapping to a genome/total # reads in the total metagenome. For the mapped reads, a breadth of >0.5 was used as QC. Breath (or breadth of coverage) is a measure of how much of a region is covered by sequencing reads (discussed further in https://instrain.readthedocs.io/en/latest/overview.html), giving an approximation of how well the reference sequence being used is represented by the reads. Shannon diversity was calculated based on the percent of mapped reads. For detailed information on profiling using MiFoDB, visit: https://mifodb.readthedocs.io/

### Comparative profiling

Fermented food samples were profiled against Kraken2 (11), MetaPhlAn4 (9), and Kaiju (10) (Table S6b through d). For Kraken2, all trimmed reads were profiled using Kraken2 v2.0.8-beta. For MetaPhlAn4, all trimmed reads were profiled using MetaPhlAn v4.0.6. For Kaiju, all trimmed reads were uploaded to Kbase (https://www.kbase.us/), and profiled using Kaiju v1.9.0.

### Characterization of unmapped reads

After installing sylph 0.5.1 (23), reads from all trimmed fastq metagenomes were profiled against the GTDB-R214 based c200 database, allowing for higher sensitivity for low-abundance genomes. Resulting .tsv output was used for downstream analysis.

### Strain sharing analysis

Strain tracking was performed using inStrain compare (inStrain v1.8.0 [13]). Output included a distance matrix for each species with popANI values used to cluster individual strains using “average” hierarchical clustering with a threshold of 99.999% popANI (representing <10 years of evolutionary distance). Strains shared between sample pairs were calculated based on this ANI. In addition, output from MetaPhlAn4 was used to run StrainPhlAn 4.0.6 (74).

### Untargeted kefir metabolomics

LC-MS was performed as previously described (75). Briefly, the same goat milk kefir time-course samples sequenced in this study (Table S1) were diluted at 1:10 in HPLC-grade water and spun at 300 × *g* for 5 min. The supernatant was used for downstream processing, as previously described (75). For metabolite extraction, 100% LC-MS-grade methanol containing internal standard was added (1:4, vol/vol). Next, samples were incubated for 5 min at room temperature, followed by centrifugation at 5,000 × *g* for 10 min to precipitate metabolites. Samples were transferred and dried under air to evaporate the solvent, then reconstituted in a reconstitution buffer (50:50 methanol:water, vol/vol) with internal standards. Samples were then analyzed on the LC-MS instrument Agilent Q-TOF 6545 using reverse phase c18 positive mode, reverse phase c18 negative mode, and HILIC negative mode. Compound annotation was performed using MSDIAL (v3.82) and an authentic standard reference library. Area under the curve for each metabolite was normalized using the sum of the internal standards in each sample. For annotated metabolites identified across multiple modalities, only the normalized peak area of the mode with the highest signal-to-noise ratio was used in downstream analysis.

### Metabolite-strain correlation

Metabolite normalized peak area was correlated to metagenome strain abundance (# reads mapping to a genome/total # reads in the total metagenome) across all time points using the stats (version 3.6.2) package in R. Only strains identified across >50% of time points were included in the correlation analysis.

### Functional analysis

All prokaryotic genes (*n* = 1,542,157 detected genes) previously annotated with Prodigal 2.6.3 (68) were profiled against three databases: Pfam (protein families), CARD (antibiotic resistance genes), and the CAZy database (CAZymes). Pfam annotations were performed using HMMer with the commands “hmmsearch --cut_ga --domtblout --acc Pfam-A.hmm” and “cath-resolve-hits.ubuntu-20.04” against the Pfam v34.0 database. CARD annotations were performed using the command “diamond blastp -f 6 -e 0.0001k 1” against the v3.2.5 database. CAZy annotations were performed using the command “hmmscan --domtblout Delta.faa_vs_dbCAN_v11.dm dbCAN-HMMdb-V11.txt GenomeSet_delta.genes.faa ; sh/hmmscan-parser.sh Delta.faa_vs_dbCAN_v11.dm>Delta.faa_vs_dbCAN_v11.dm.ps ; cat Delta.faa_vs_dbCAN_v11.dm.ps | awk '$5 < 1e-15&&$10 > 0.35' > Delta.faa_vs_dbCAN_v11.dm.ps.stringent” with the dbCAN v11 database. CAZyme substrate annotations were based on previously used definitions. Results were parsed using inStrain parse_annotations (inStrain v1.8.0 [13]). More detailed instructions on gene annotation and parsing can be found at https://instrain.readthedocs.io/en/latest/user_manual.html#gene-annotation.

## Data Availability

All databases are available at https://zenodo.org/records/13830159. The raw sequencing data for this study are available at NCBI under Sequencing Read Archive (SRA) accession no. PRJNA1092721 and BioProject accession no. PRJNA1092721 (https://github.com/elisacaffrey/MiFoDB).

## SUPPLEMENTAL MATERIAL

Supplemental Figures and Notes msystems.00141-25-s0001.pdf Notes S1 and S2; Figures S1 to S8.

Supplemental Tables msystems.00141-25-s0002.xlsx Tables S1 to S10.

ASM does not own the copyrights to Supplemental Material that may be linked to, or accessed through, an article. The authors have granted ASM a non-exclusive, world-wide license to publish the Supplemental Material files. Please contact the corresponding author directly for reuse.
