# Supplementary material for: MiFoDB, a workflow for microbial food metagenomic characterization, enables high-resolution analysis of fermented food microbial dynamics
Source: mSystems. 2025 Aug 19;10(9):e00141-25. doi: 10.1128/msystems.00141-25 (PMC12456020; doi:10.1128/msystems.00141-25)
Supplement: Supplemental Figures and Notes — Notes S1 and S2; Figures S1 to S8. [file msystems.00141-25-s0001.pdf]

Supplementary Note 1

An important consideration in profiling metagenomic data is the presence of unmapped reads. Species with completely novel genomes, such as the 45 mentioned in the main text (see *Generation of the MiFoDB genome database*) (i.e., with previously unidentified genomes) present in a given food are one source of unmapped reads. A second source of unmapped reads could be due to species with known genomes (i.e., in GTDB) that are currently not in our database. To address this second scenario, we profiled all our samples against GTDB[1] using sylph[2], a metagenomic profiling program. Profiling resulted in the identification of 1068 unique genomes, 338 of which were not included in MiFoDB and were identified in more than one sample or with a reported abundance > 0.5%. We created a version of MiFoDB (MiFoDB_beta_v3) that incorporated these genomes from NCBI, and our 89 samples were profiled against it (Supplementary Fig. 3b). Profiling results showed an average increase in mapped reads of ~2.6% (65.2% ± 2.12% SEM high confidence reads mapping using MiFoDB_beta_v3, compared to 62.6% ± 2.08% SEM high confidence reads mapping using MiFoDB_beta_v2). Due to the small resulting change in abundance of mapped reads, and that the majority of newly added genomes mapping >1% were only identified in one sample each, we chose to not include these genomes for downstream analysis. However, use of sylph to identify and incorporate missing genomes to the core database could aid certain applications of MiFoDB, such as in the mapping of understudied ferments. MiFoDB_beta_v3 has been made available on Zenodo.

References

1. Parks, D. H. *et al.* GTDB: an ongoing census of bacterial and archaeal diversity through a phylogenetically consistent, rank normalized and complete genome-based taxonomy. *Nucleic Acids Res.* **50**, D785–D794 (2022).

2. Shaw, J. & Yu, Y. W. Metagenome profiling and containment estimation through abundance-corrected k-mer sketching with sylph. *bioRxiv* 2023.11.20.567879 (2024) doi:10.1101/2023.11.20.567879.

Supplementary Note 2

A. oryzae is a Generally Regarded As Safe (GRAS) filamentous fungus used in production of rice and soy based ferments like miso, soy sauce, and amazake. Closely related *A. flavus* and *A. parasiticus* are pathogenic, known to infect seed crops and produce aflatoxin, a carcinogenic secondary metabolite[1]. A similar parallel might be drawn to *E. coli* and *Shigella*: while genetic relatedness would classify *E. coli* and *Shigella* as a single species, due to clinical significance the two microbes retain distinguished classifications, and research has focused on identifying accurate and reliable markers to distinguish the two[2]. While no *A. oryzae* strain has been reported to produce aflatoxin on rice and soy substrates, it retains an aflatoxin biosynthesis gene cluster[3].

We expanded our search for identifying markers of *A. oryzae* and *A. flavus* by including samples of various ferments made with rice koji inoculated with commercial *A. oryzae* as controls. Samples were mapped to *A. flavus* and *A. oryzae* RefSeq genomes, with all multi-mapped reads removed. Scaffolds in all samples mapped to *A. flavus*, including samples made with a commercial rice koji starter. As it is highly unlikely that *A. flavus* is present in samples made with koji from commercial *A. oryzae*, identification of *A. flavus* indicates a high false positive rate (Supplementary Fig. 6). In keeping with the goal of this database to provide information about microbes involved in food fermentation, MiFoDB_euk does not include *A. flavus*. In order to confirm that our samples do not contain *A. flavus,* genomes from a number of Aspergillus species including 7 different *A. flavus* references and 4 *A. oryzae* genomes were clustered with bins of interest using Mash[4]. Resulting dendrogram shows formation of two sub-clades, one containing *A. oryzae* reference genomes and fermented food sample bins (average ANI to sample bins = 0.99559931), and the other containing only *A. flavus* reference genomes (average ANI to sample bins = 0.99180935) (Supplementary Fig. 7). While the distinct clustering of the two species genomes (reference genomes and MAGS) offers support for the lack of *A. flavus* in the novel superior qu sample, it remains important to use caution when classifying *A. oryzae* and *A. flavus*, particularly in novel fermented food samples. Out of abundance of caution, coupling of MiFoDB-profiled metagenomics with HPLC methods to detect aflatoxin presence would allow for the highest degree of certainty when it comes to characterizing novel *Aspergillus*-based fermented foods.

References

1. Amaike, S. & Keller, N. P. Aspergillus flavus. (2011) doi:10.1146/annurev-phyto-072910-

095221.

2. Parks, D. H., Chuvochina, M., Reeves, P. R., Beatson, S. A. & Hugenholtz, P. Reclassification of Shigella species as later heterotypic synonyms of Escherichia coli in the Genome Taxonomy Database. *bioRxiv* 2021.09.22.461432 (2021) doi:10.1101/2021.09.22.461432.

3. Payne, G. A. *et al.* Whole genome comparison of Aspergillus flavus and A. oryzae. *Med. Mycol.* **44**, S9–S11 (2006).

4. Ondov, B. D. *et al.* Mash: fast genome and metagenome distance estimation using MinHash. *Genome Biol.* **17**, 132 (2016).

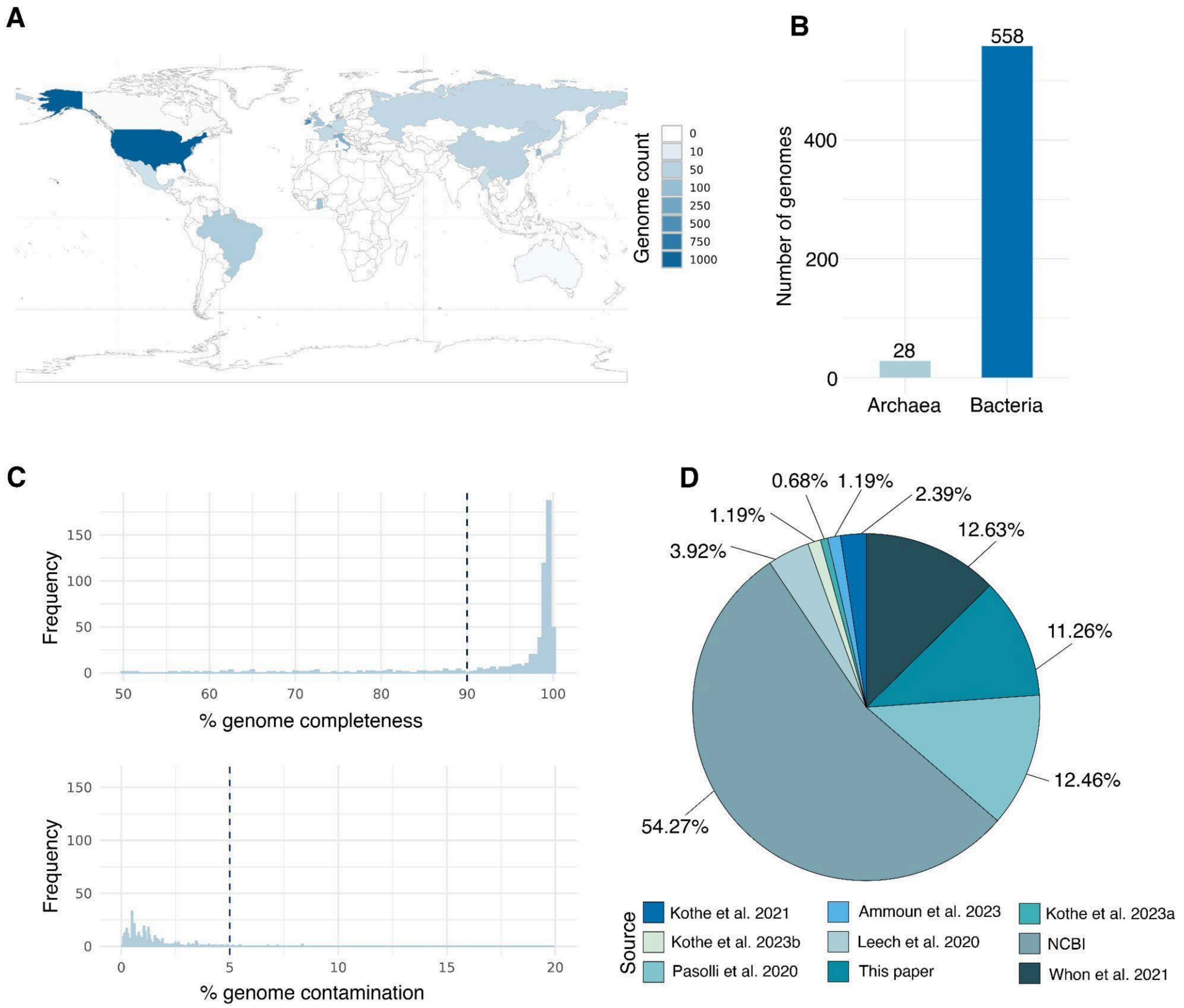

Supplementary Figure 1

A. Map of sample origin countries; genome count indicated by color intensity.
B. Frequency of archaea and bacteria in MiFoDB_prok.
C. Completeness and contamination scores from dRep for all prokaryotes in MiFoDB_prok. >90% completeness and <5% contamination are marked with dotted lines.
D. Contribution of each input dataset to MiFoDB_prok.

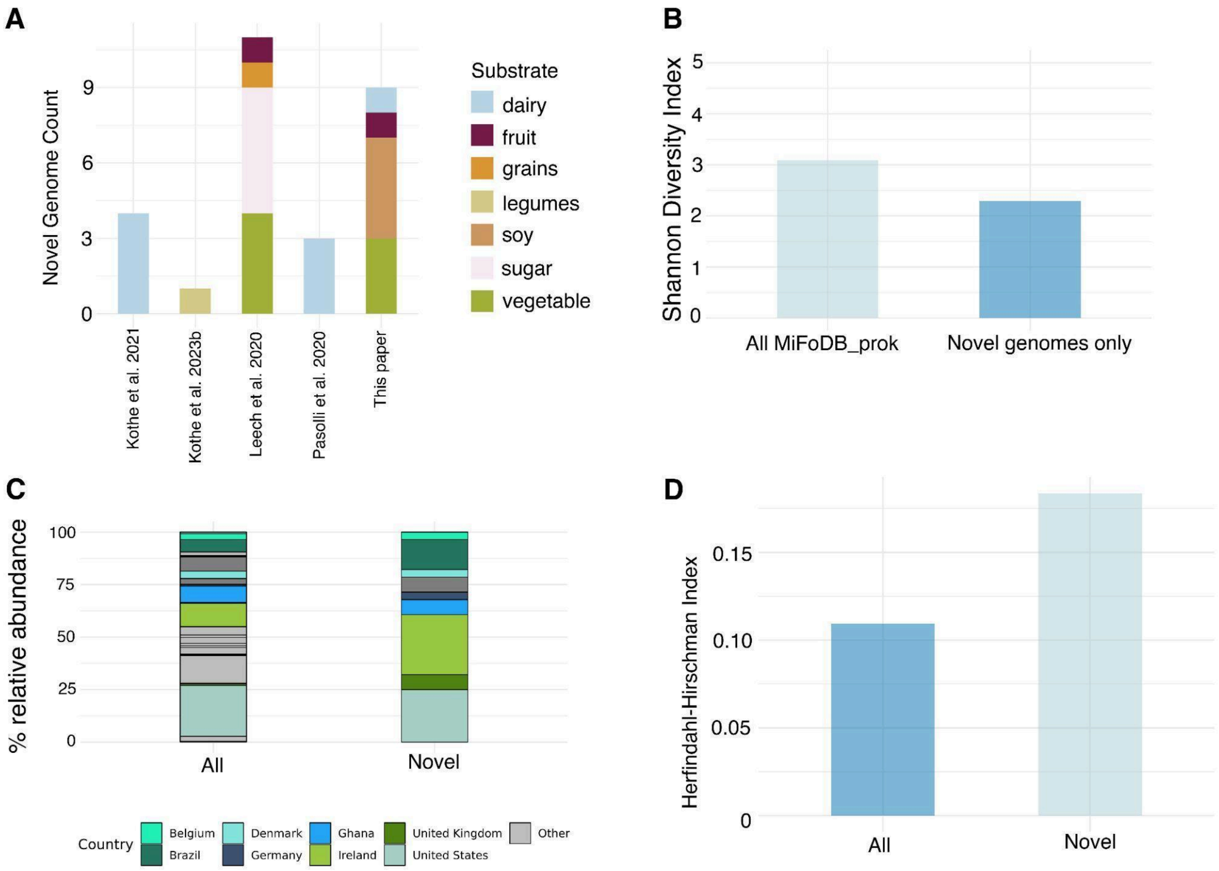

Supplementary Figure 2

A. Novel genomes in MiFoDB_prok contributed by individual studies, with the substrate of origin indicated by color.

B-D. Three metrics of diversity for all genomes and novel genomes in MiFoDB_prok; Shannon Diversity (B); relative abundance by country of origin (C); and the Herfindahl index (D), where a higher score reflects a smaller number of countries representing the majority of novel genomes.

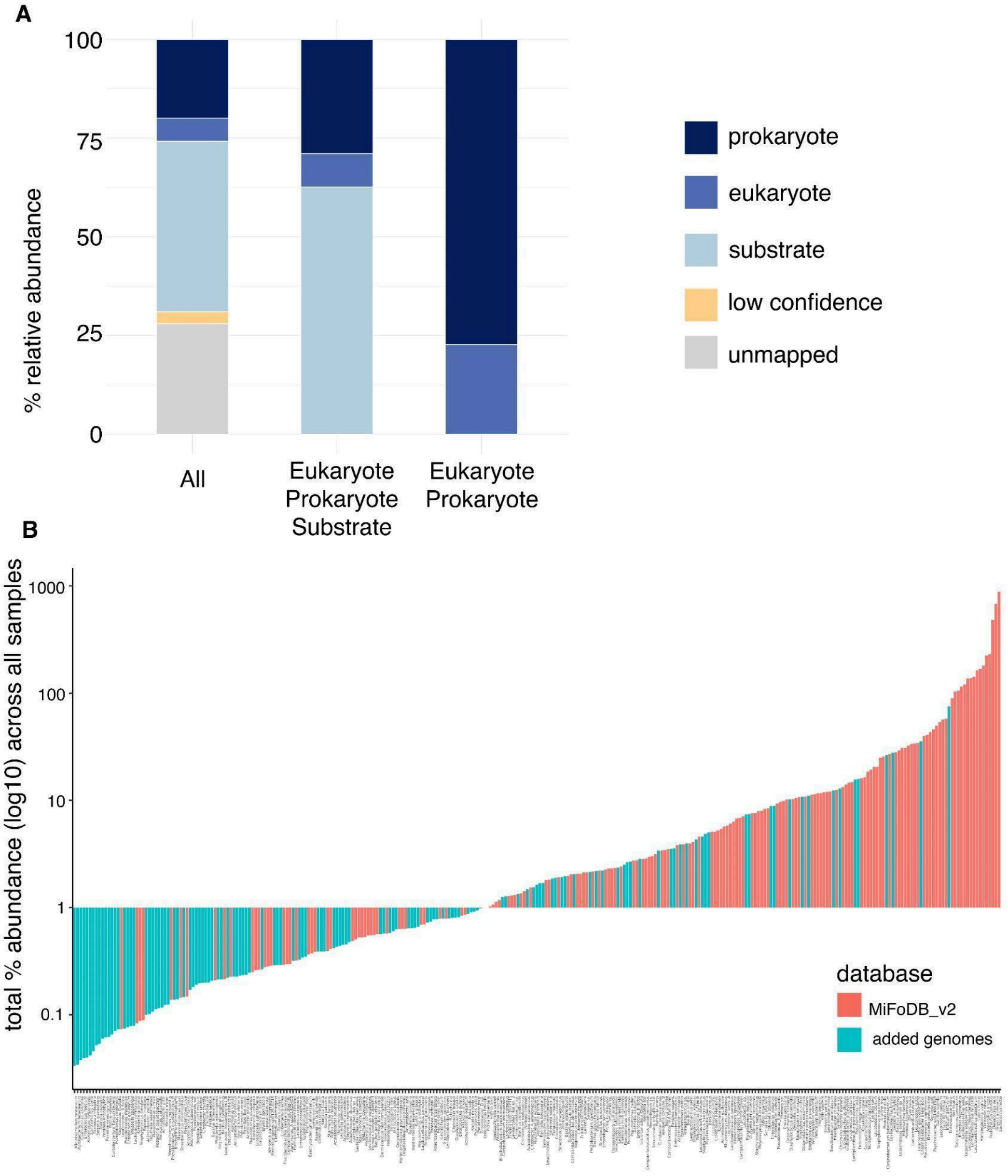

Supplementary Figure 3

A. Relative abundance of metagenomic reads mapping to each database for pikliz, a Haitian vegetable ferment. Use of all databases gives the most accurate representation of the sequenced data, including low confidence and unmapped reads. By only using mapped reads (Eukaryote Prokaryote Substrate) the data becomes skewed so that the ratio of all prokaryotes, eukaryotes and substrate increase, when in reality over 25% of the sample remains unmapped. When only using the prokaryote and eukaryote database, prokaryote reads which previously represented ~30% of the sample now make up >75%.
B. Total mapped abundance of each genomes identified across all 90 samples. Genomes in MiFoDB_v2 are in red, while genomes added after identification in sylph that were not in the original dataset (MiFoDB_v3) are in blue. The majority of novel genomes (in blue) make up less than 1% of the total abundance across all 90 samples. Mapped abundance is shown using a log10 scale in order to better visualize the abundance of genomes making up <1% of total abundance.

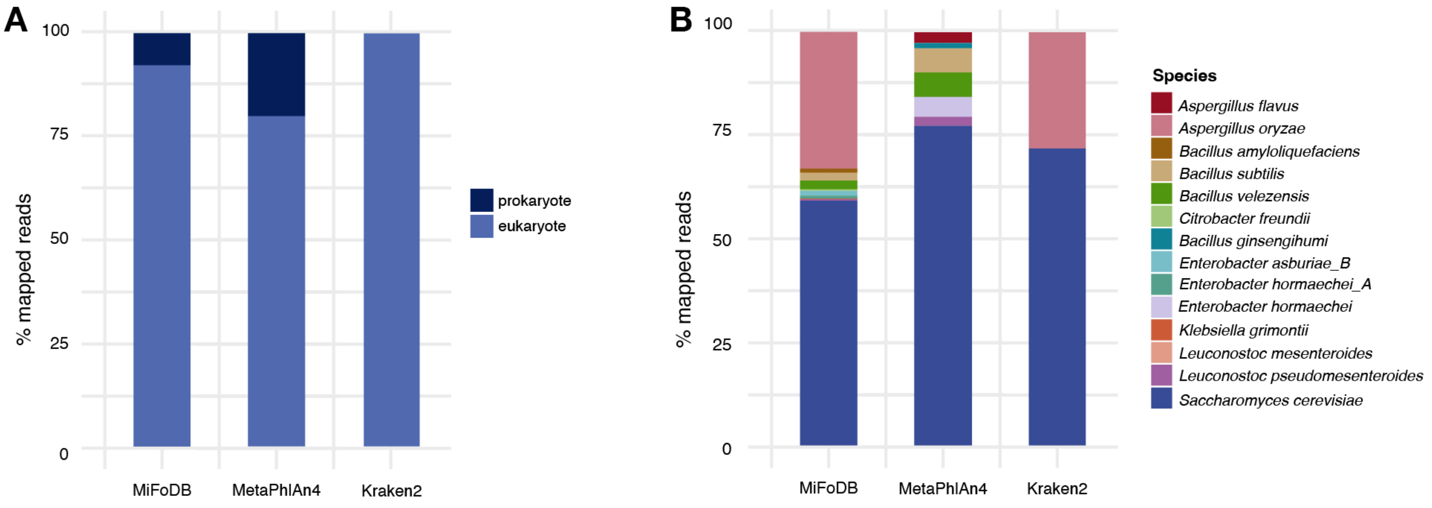

Supplementary Figure 4

A. Comparison of the percent of mapped reads using MiFoDB, MetaPhlAn4 and Kraken2 (Supplementary Table 6a-b,d). Low confidence reads were removed from MiFoDB, and mapped reads were scaled to 100% in order to compare to MetaPhlAn4 and Kraken2. Low abundance reads (<1%) were filtered from MetaPhlAn4 and Kraken2 for comparative QC. While MiFoDB and MetaPhlAn4 did report both prokaryotes and eukaryotes, Kraken2 only reported eukaryotes.

B. Percent of high confidence mapped reads (breadth > 0.5 for MiFoDB and abundance > 1% for MetaPhlAn4 and Kraken2) by species. Kraken2 failed to report any prokaryotes in the sample (Fig. 3c), only identifying *Saccharomyces cerevisiae* and *Aspergillus oryzae*. MetaPhlAn4 did report both prokaryote and eukaryote genomes, showing a dominance of *Saccharomyces cerevisiae*, as well as *Aspergillus flavus*, with no *Aspergillus oryzae* reported.

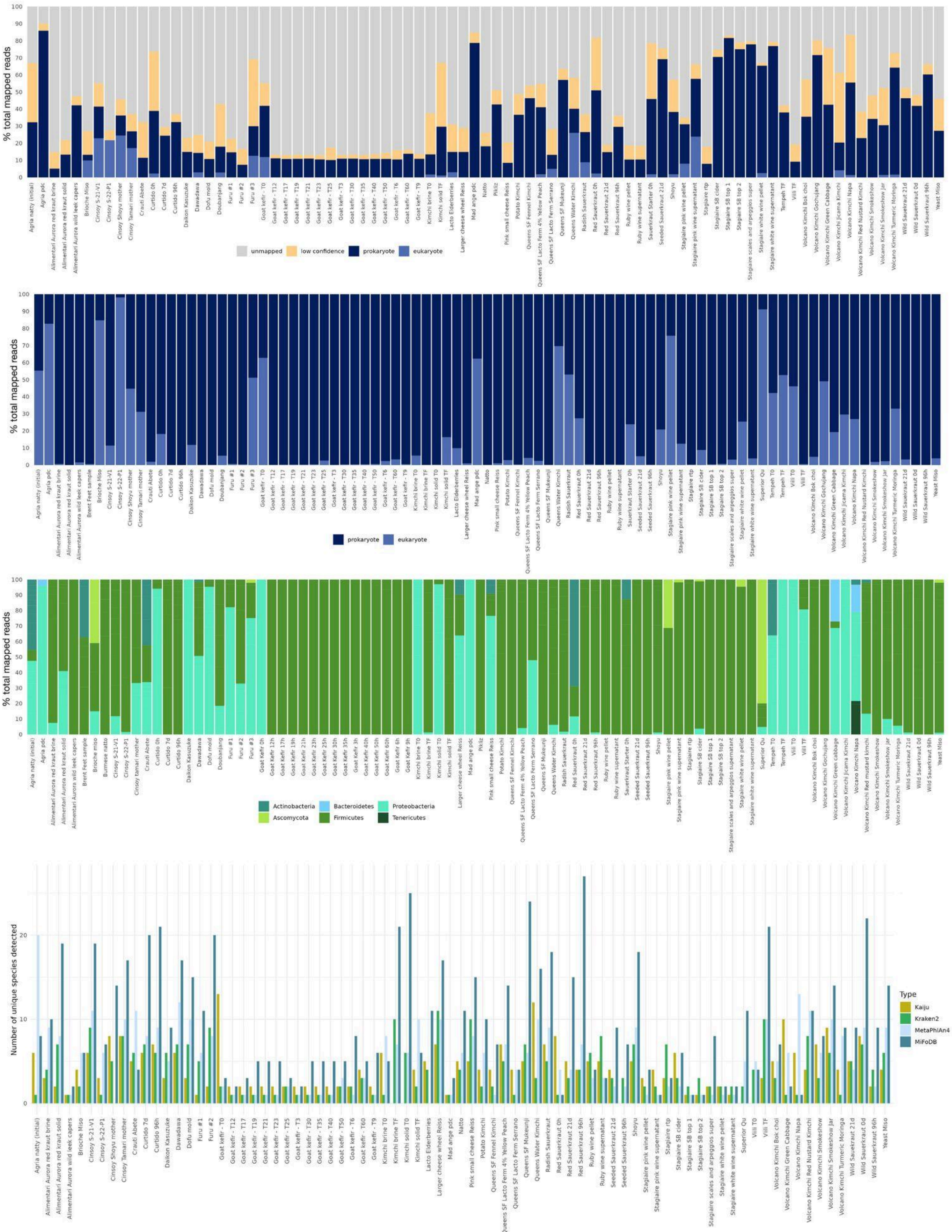
A
% total mapped reads
unmapped
low confidence
prokaryote
eukaryote
B
% total mapped reads
prokaryote
eukaryote
C
% total mapped reads
Actinobacteria
Bacteroidetes
Proteobacteria
Ascomycota
Firmicutes
Tenericutes
D
Number of unique species detected
Type
Kaiju
Kraken2
MetaPhlAn4
MiFoDB

Supplementary Figure 5

A. **Profiling results with Kaiju:** Mapped reads were categorized as "low confidence" when the species abundance was <1%. High confidence mean reads mapped were almost half of MiFoDB = 31.7% ± 2.41% SEM). Only 84 samples were profiled due to repeated Kbase preprocessing failure.
B. **Profiling results with Kraken2:** Percentage of mapped reads that passed standard QC threshold of 1% species abundance). Kraken2 reports mapped read percentages, making it challenging to understand which percentage of reads in the sample remain unmapped. Across all 89 samples, Kraken2 identified 194 unique species; 199 unique species were identified using MiFoDB (Figure 2A, Supplementary Table 6d).
C. **Profiling results with MetaPhlAn4:** Percentage of mapped reads that passed QC (>1% species abundance). MetaPhlAn4 reports mapped read percentages, making it challenging to understand which percentage of reads in the sample remain unmapped. As the results only show prokaryote mapping, prokaryotes are represented by phyla. Across all 89 samples, MetaPhlAn2 identified 191 unique species; 199 unique species were identified using MiFoDB (Figure 2A, Supplementary Table 6b).
D. **Comparison of high confidence unique species detected using MiFoDB, MetaPhlAn4, Kaiju, and Kraken2:** Following QC (breadth > 0.5 for MiFoDB, or abundance >1% for Kraken2, Kaiju, and MetaPhlAn4), the number of unique species identified was compared. On average, MiFoDB detected 8.6 species, with 5.3, 3.8. and 4.5 identified using MetPhlAn4, Kraken2, and Kaiju, respectively (Supplementary Table 6a-d).

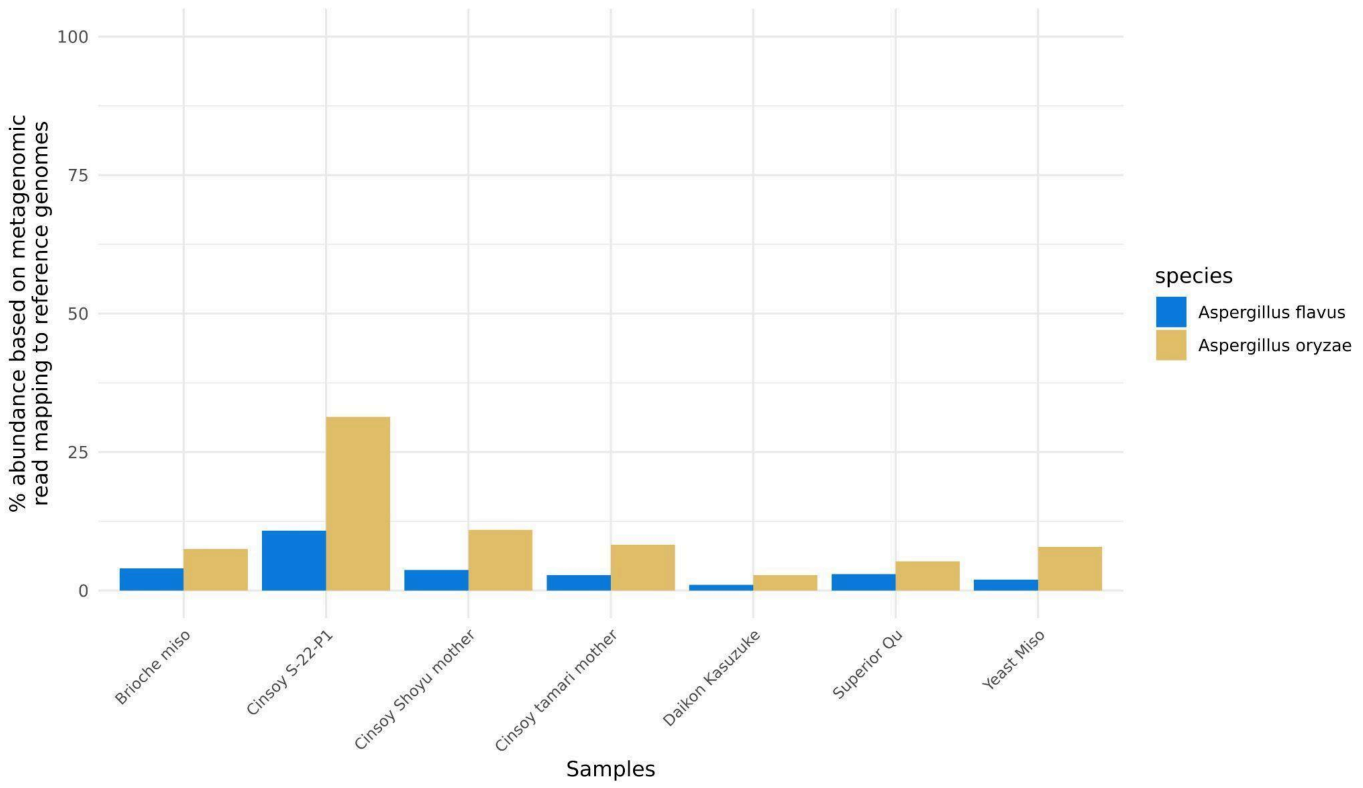

Supplementary Figure 6

Abundance of metagenomic reads mapped to *A. flavus* and *A. oryzae* in indicated samples. All samples excluding Superior qu and radish sauerkraut are highly unlikely to contain *A. flavus*, as they were produced using commercially available rice koji and subject to food safety testing (e.g., aflatoxin); furthermore, phylogenetic analysis (See Supplementary Fig. 6) indicates the *A. flavus* mappings are false positives reflecting the high identity between genomes.

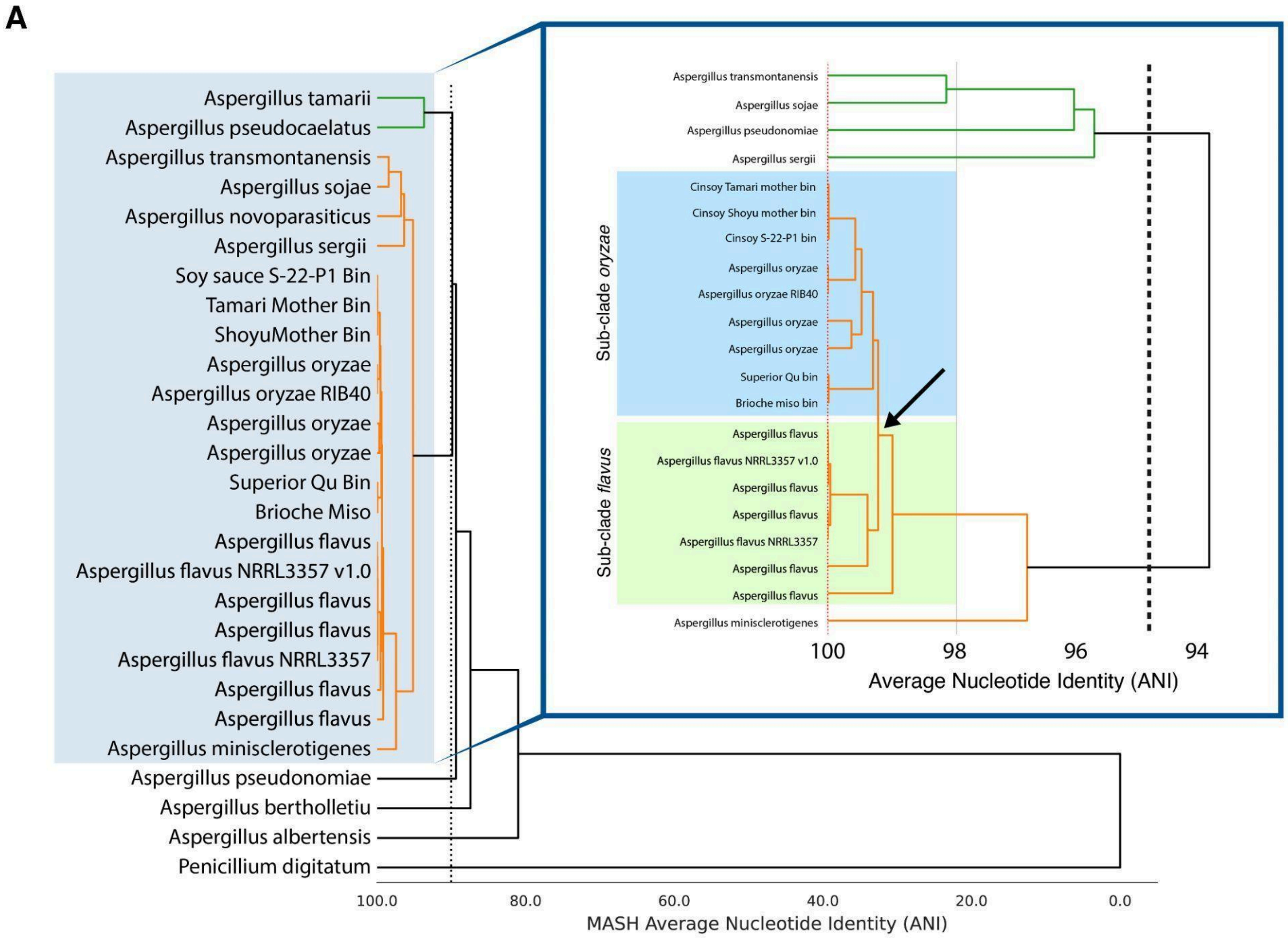

Supplementary Figure 7

MASH clustering of reference genomes for *A. oryzae* and *A. flavus*, along with MAG of interest. The blue box shows an expanded dendrogram of genomes within a 95% ANI of each other, with two subclades highlighted: *A. flavus* sub-clade (green) and *A. oryzae* sub-clade (blue). The arrow indicates the node of separation between these clades. All samples from our fermented foods, including those produced commercially cluster with *A. oryzae* and superior qu.

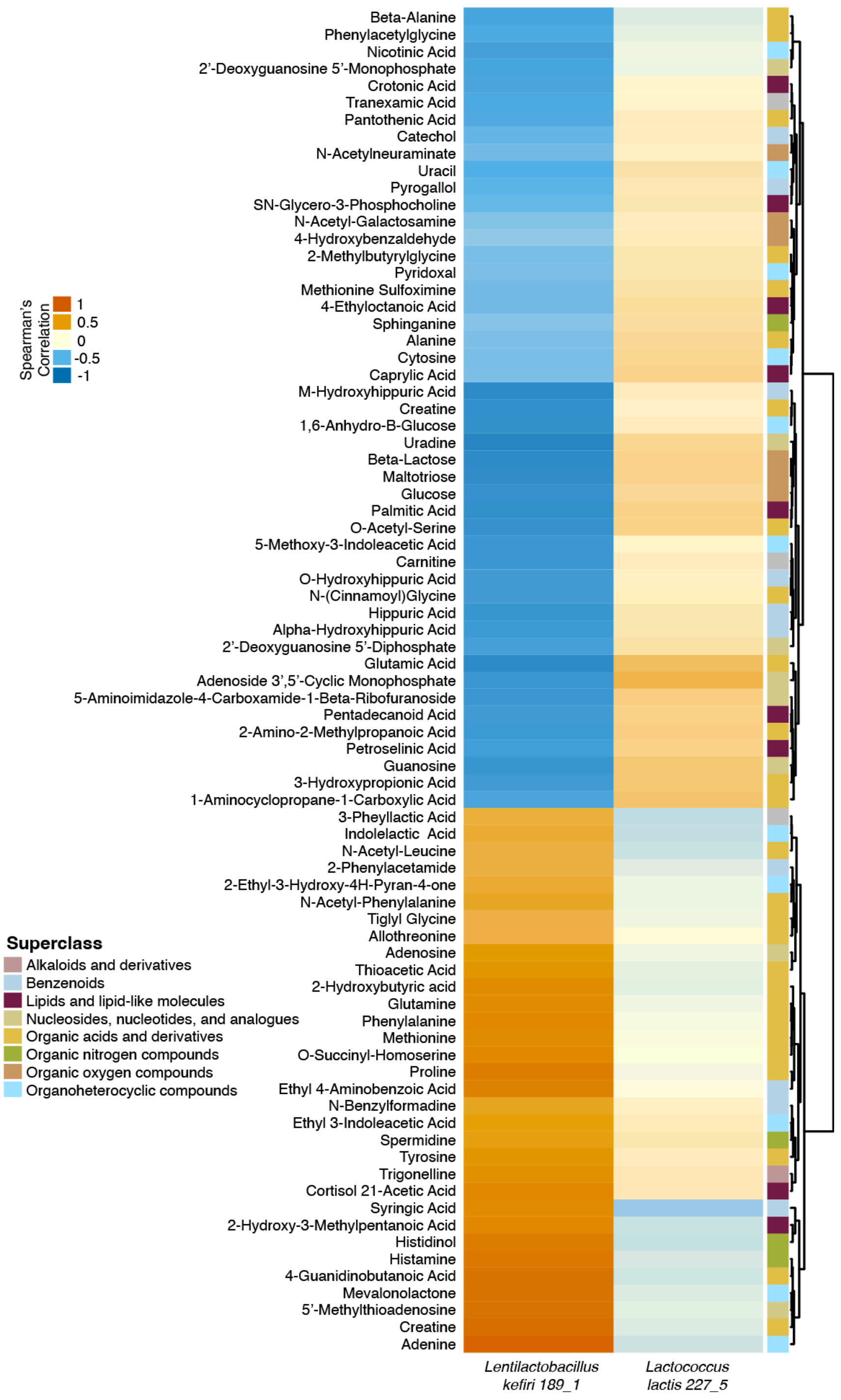

Supplementary Figure 8

Spearman correlation of strain abundance to semi-targeted LC-MS results across 14 timepoints of goat kefir fermentation. A full list of metabolites is available in Supplementary Table 9.
